# Mismatch negativity–stimulation paradigms in past and in future

**DOI:** 10.3389/fnins.2022.1025763

**Published:** 2022-11-17

**Authors:** Mari Tervaniemi

**Affiliations:** ^1^Center of Excellence in Music, Mind, Body, and Brain, Faculty of Educational Sciences, University of Helsinki, Helsinki, Finland; ^2^Cognitive Brain Research Unit, Department of Psychology and Logopedics, Faculty of Medicine, University of Helsinki, Helsinki, Finland

**Keywords:** music, audition, musical learning, cognition, EEG, fMRI

## Abstract

Mismatch negativity (MMN) studies were initiated as part of a well-controlled experimental research tradition with the aim to identify some key principles of auditory processing and memory. During the past two decades, empirical paradigms have moved toward more ecologically valid ones while retaining rigid experimental control. In this paper, I will introduce this development of MMN stimulation paradigms starting from the paradigms used in basic science and then moving to paradigms that have been particularly relevant for studies on music learning and musical expertise. *Via* these historical and thematic perspectives, I wish to stimulate paradigm development further to meet the demands of naturalistic ecologically valid studies also when using MMN in the context of event-related potential technique that necessarily requires averaging across several stimulus presentations.

## Introduction

Thanks to versatile development in theoretical and methodological domains, auditory cognitive neuroscience has witnessed immense progress in past decades. When considering the development of methodology in the field, the main emphasis of scientific discussion is commonly given on methods in data acquisition and analyses. However, when considering the key questions of the field (specifically brain basis underlying neuroplasticity particularly in the domains of auditory learning, development, and aging), it is evident that validity of the stimulation paradigms is also of utmost importance. If these paradigms (that is, their sounds and the auditory soundscapes created by them) fail to address the neurocognitive processes of interest, the results are of minimal use in scientific or applied perspectives.

Notably, while a transition from well-controlled laboratory-based studies toward ecologically valid stimulation and recording paradigms has occurred in several related research traditions such as social and emotion neuroscience, it is questionable whether this is a feasible framework for studies in auditory cognitive neuroscience, particularly when event-related potential (ERP) technique and the mismatch negativity (MMN) are of interest. This perspectives paper aims to offer a framework for observing the development of stimulation paradigms of the MMN field since the 1970s and to propose some future novel advancements. The discussion will be divided into two main sections, the first on basic MMN studies and the second on MMN studies in music-related contexts. After them, the brain generators of the MMN will be briefly illuminated. In the end of the paper, future directions of the MMN will be discussed.

## Historical overview on mismatch negativity studies in oddball and multi-feature paradigms

When pioneering studies that launched mismatch negativity (MMN) were conducted (Näätänen et al., 1978), the fundamental question of the highest theoretical relevance was actually quite simple: is it possible to isolate a difference signal from the human brain? In other words, is there a neural signal that can differentiate acoustically different frequent standard and rare deviant sounds from each other? At that time, EEG recording and sound stimulation technologies were rather limited, and studies were conducted using sinusoidal sounds in an oddball paradigm. Once MMN had been established as a general index of the difference monitoring and sensory memory, empirical studies were conducted to indicate those sound parameters that are encoded in the sensory memory (e.g., Paavilainen et al., 1993 for duration, and Näätänen et al., 1987 for intensity). Further, parametric studies were conducted to indicate the accuracy of the sensory memory in this encoding (Sams et al., 1985 for frequency) and the correspondence between the MMN parameters and perceptual accuracy (Tiitinen et al., 1994 for frequency; Amenedo and Escera, 2000, for duration).

**FIGURE 1:**
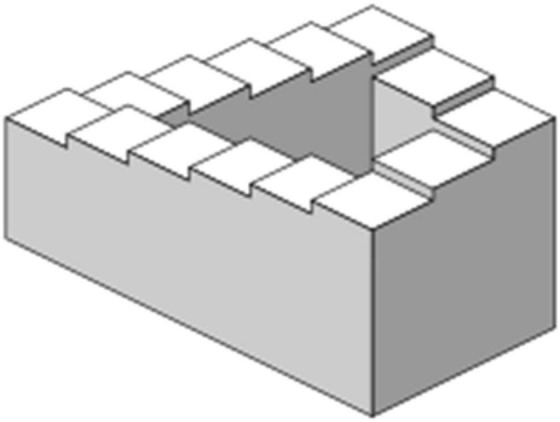
Visual analogue (endless Penrose stairs) of ever ascending/descending sound sequence created by Shepard (reproduced from Wikipedia).

The next generation of studies aimed to avoid the co-occurrence of acoustical deviance and rareness of the deviant stimulus. This may sound simple, but it is less so since perceptual deviance is most often coupled by acoustical features. The solutions were diverse. First, Yabe et al. (1997) and Tervaniemi et al. (1994a) used *sound omission* as the deviant stimulus in isochronous sequences and in tone pairs, respectively. They both showed that MMN can be generated by a sound omission but only within a definite window enabling integration of incoming auditory information for some hundreds of milliseconds only. Second, Winkler et al. (1995) used a phenomenon called *missing fundamental* that denotes an “illusion” of the sound’s fundamental frequency being identified even if this specific frequency is not present in the sound at all; it is computed in the brain based on the spectrum of the harmonic overtones. They showed that the MMN indeed reflects perceived fundamental frequency that can be created by several combinations of overtones, while a subset of the same overtones in a different constellation causes a perception of a different fundamental frequency and, subsequently, the MMN. Third, Tervaniemi et al. (1994b) utilized another auditory illusion created by Shepard tones. They can be presented in an ascending or descending manner in a loop to give an impression of *an endlessly ascending or descending pitch* (Figure 1). In the MMN experiment, these Shepard tones were looped to create an illusion of continuous pitch decrement that was eventually interrupted by a pitch repetition or by an ascending pitch. It was found that both pitch repetition and ascending pitch evoked the MMN when using Shepard tones. This was taken as evidence of the MMN being an index of violated prediction of the pitch of the sound-to-come rather than an index of sensory memory representation only.

Despite the theoretical relevance of the paradigms mentioned above, they had less to offer for applications of the MMN in clinical studies or studies with child participants. In traditional oddball paradigms, one sequence had one or maximally three deviants, making the studies rather long and repetitive, particularly if the signal-to-noise ratio was to be optimized by maximizing the number of sound presentations. As a solution, Näätänen introduced the idea of having several deviants in one sequence with one standard. Here, the basic assumption is that a standard sound is encoded as a sum of its acoustic features. Thus, one deviant can differ from this standard “template” independently by one or several features, as shown by so-called additivity studies by Schröger (1995) in which the MMN parameters sensitively reflected the number of violated sound features. When MMN recorded in a traditional oddball paradigm was compared with an MMN recorded in this multi-feature paradigm, there was no significant difference in the MMN parameters (Pakarinen et al., 2010). However, the recording time was remarkably shorter and thus the MMN recordings became more feasible with many clinical populations and in children.

## Mismatch negativity paradigms in music-related studies

**FIGURE 2:**
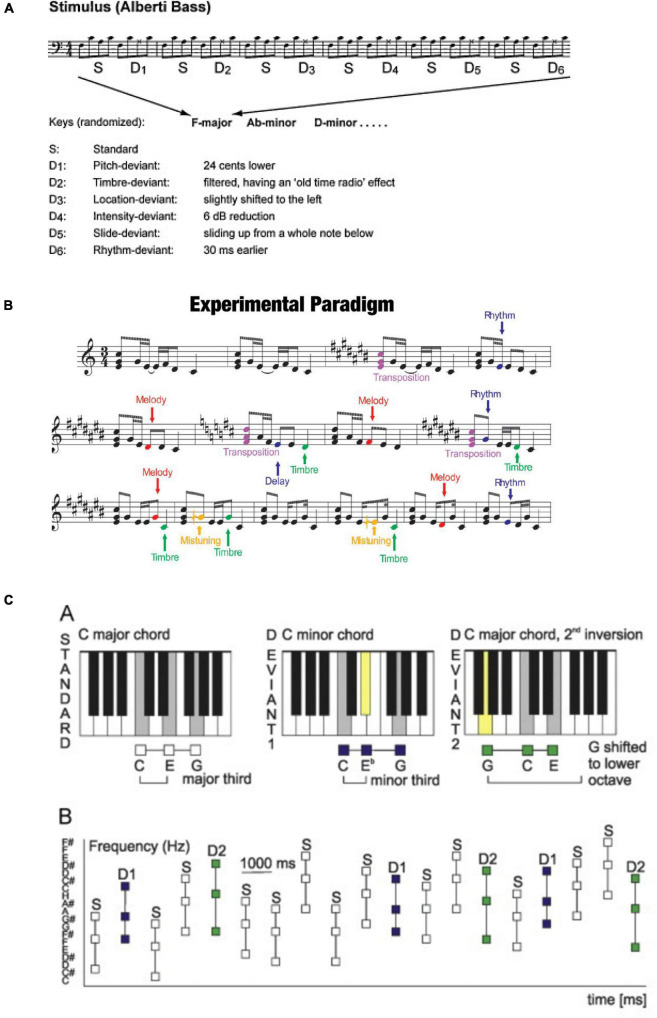
**(A)** Musical multifeature paradigm that includes sound patterns with six different deviant tones as indicated below the score. The sequence is presented in one key for six bars and then transposed to a new key, in other words, it was presented at various pitch levels. Reprinted from Vuust et al. (2012) with permission from Elsevier. **(B)** Melodic multifeatured paradigm that includes short melodies with three different acoustic deviances and three different cognitive deviances as indicated on the right. Cognitive deviants change the content of the melody while acoustic deviants do not. One of the cognitive deviants is transposition, meaning that the melody is presented at various pitch levels. Reproduced by permission from Tervaniemi et al. (2014). **(C)** Chord paradigm with standard and two different deviant chords as indicated in the upper row. During the experiment, these three chords are presented at randomly varying pitch levels (reproduced from Virtala et al. (2014) under CC-BY license).

Based on the paradigm development described above, about 15 years ago interests emerged to develop “musical” MMN stimulation paradigms to probe the neural basis of musical skills. The first of these paradigms was based on the idea about multiple acoustical features being encoded in parallel and thus being behind the generation of the MMN. In the group of prof. Vuust, the starting point was an Alberti bass—a looped sound sequence often used in the classical era as an arpeggio. There, the sounds of a given triad chord were presented in the order “lowest, highest, middle, highest” in a looped manner (Vuust et al., 2012; Figure 2A). In this paradigm, the recording time was less than 15 min for a total of six different deviants, thus data collection is considerably faster than in traditional paradigms. In the melodic multi-feature paradigm developed by prof. Huotilainen, a looped 2-s melody was used as the starting point (Putkinen et al., 2014; Figure 2B). This melody also included a total of six deviants, three of which modulated the structure of the melody for its successive presentations. The data collection here also took less than 15 min.

By employing these musical multi-feature paradigms, it was shown that the MMN reflects the musical expertise and their participant background in a genre-specific manner; the sound parameters that are most important in a performance of a given musician evoke the largest MMN, P3a response, or both [for a review, see Putkinen and Tervaniemi (2018)]. The MMN was also shown to emerge in a gradual feature-specific manner during music training in children learning to play an instrument during their school years from 9 to 13 years of age (Putkinen et al., 2014). Furthermore, implicit vs. explicit forms of expertise were shown to have different neural trajectories as reflected by the MMN; while enthusiastic jazz listeners had a diminished MMN to a slide deviant, professional jazz performers showed an enlarged MMN to this deviant and to timbre and pitch deviants (Kliuchko et al., 2019). Thus, these paradigms highlighted the complexity of music learning and have also been helpful in differentiating implicit and explicit profiles in music listeners vs. performers.

In addition to looped melodic and chordal paradigms, various MMN studies have also been conducted using randomized chord sequences consisting of two or more triad chords (e.g., major chords as standards and minor chords as deviants). These studies have been conducted using several paradigms and there is no paradigm we could nominate as the prevalent paradigm (unlike in looped musical paradigms). Here, the first paradigms only used two chords and thus had the co-occurrence of acoustic and musical deviance; major and minor chords were different from each other in both manners [Tervaniemi et al., 1999; Brattico et al., 2009; and Tervaniemi et al., 2011 with magnetoencephalography (MEG) and Tervaniemi et al., 2000 with positron emission tomograpy (PET)]. More recently, Virtala et al. (2011) with EEG; Figure 2C created a paradigm in which the contribution of acoustical deviance could be excluded. This was accomplished by creating the stimulus chords from various tones at several frequency levels. By this design it was possible to control and balance how often each tone was presented either as part of a major chord or as part of a minor chord. Thus, any difference in the MMN evoked by the chords was a result of its category (major/minor) and not its acoustical composition. Using this chord-MMN paradigm, it was observed that already newborn infants can differentiate major and minor chords from each other (Virtala et al., 2013) and that music training enhances this differentiation in adolescents and in adults (Virtala et al., 2012, 2014).

In addition to major/minor mode, another dimension of any musical interval or chord is its consonance or dissonance. This attribute is often reduced as the pleasantness and unpleasantness of the intervals or chords, respectively, even if this nomenclature is not accurate since some individuals prefer dissonant “unpleasant” intervals, chords, and music excerpts over consonant “pleasant” intervals (see next paragraph). To investigate the effects of musical expertise on consonance/dissonance discrimination, Linnavalli et al. (2020) created two types of dissonant chords and introduced them in the context of consonant chords. They included groups of professional musicians and non-musicians as their participants. It was found that both groups of participants discriminated dissonant chords from consonant ones both neurally and behaviorally. In the behavioral task, the musicians were more accurate than the non-musicians without a group difference in the MMN elicitation. As the dissonant chords elicited MMN responses for both groups, sensory dissonance seems to be discriminated in an early sensory level, irrespective of musical expertise, and the facilitating effects of musical expertise for this discrimination seems to be activated only in later stages of auditory processing, as reflected by performance in the behavioral auditory task.

As the last example of the use of MMN in music-related studies, a recent paradigm developed by Sarasso et al. (2022) will be introduced. Sarasso and colleagues used intervals of two kinds: consonant (perfect fifth) and dissonant (tritones) at low and high frequency levels. The novel aspect in their study is that the data were analyzed based on the participants’ preference for these intervals; half of them preferred consonant intervals, half of them dissonant intervals. It was found that irrespective of the acoustical and musical characteristics of the intervals, it was the most preferred and “attractive” interval that evoked larger MMN when compared with the other, less attractive interval. Moreover, computational Bayesian surprise index was associated with both MMN and behavioral indices, suggesting that (early) auditory learning is related to higher-order aesthetic processing of music sounds.

## Mismatch negativity generators

Main contribution to the scalp recorded auditory MMN originates from the auditory cortices with an additional generator in the right frontal lobe [for a review, see Näätänen et al. (2010); see below]. Important in the current context is to note that the MMN generator source within the auditory areas may also vary as a function of the stimulus complexity: when an identical pitch change was embedded in an oddball sequence of sinusoidal tones versus musical chords, the MEG recordings indicated the MMN generator to be more medially located when more complex (musical) stimuli were used (Alho et al., 1996). Furthermore, in non-musicians, the left vs. right auditory cortices may adopt different roles as a function of the stimulus type: in PET and MEG experiments, the left auditory areas responded more strongly to changes in phonemes (Tervaniemi et al., 2000) and rhythm (Vuust et al., 2005) while the right auditory areas respond more strongly to changes in chords (Tervaniemi et al., 1999, 2000). However, this asymmetry may also be modulated by musical expertise: musicians were found to have predominantly left-hemispheric (MEG counterpart of) MMN to chord changes (Tervaniemi et al., 2011).

In addition to the auditory areas, also frontal areas, particularly the right inferior frontal gyrus, can be activated by the deviants when presented in an oddball paradigm, at least when the stimulation has acoustically small deviances (Opitz et al., 2002). Recently, using the melodic multifeature paradigm, it was shown that while the sensory deviants (e.g., timbre) were primarily processed in the auditory areas, the cognitively more demanding deviants (e.g., transposition) were primarily processed in the frontal areas (Bonetti et al., 2022). Together, these findings point to the multifaceted characteristics of the MMN generation along the sensory-cognitive-axis of our auditory neurocognition and, respectively, in the brain.

Finally, the deviance detection as indexed by the MMN may be initiated already below cortical areas. This was shown by fMRI findings using naturalistic stimuli (pseudoword/ba:ba/and its close acoustical musical counterpart produced by saxophone) in a semi-attend paradigm (Tervaniemi et al., 2006). There, non-musicians were instructed to indicate by a button press whether each sound was speech or music sound but not to pay attention to slight deviances. It was found that in addition to BOLD activations in the temporal and frontal areas, deviances in sound pitch and duration activated also thalamic structures. This finding is in line with increasing body of the literature highlighting the roles of ascending auditory pathways in deviance detection (Escera and Malmierca, 2014).

## Future directions

This current perspective paper sought to highlight the developments in past decades in paradigms that have been developed in MMN studies for basic science and music-related research projects (due to the space limitations of this paper, clinical studies had to be ignored despite their high relevance). Even if the MMN was originally considered as a tool for investigating learning and neurocognition of simple sounds in simple contexts, there are now several paradigms that enable investigating higher-order phenomena, such as musical development, musical expertise, and appreciation. Thus, the progress of the paradigm development(s) enables theoretical advancement that is needed in the larger field of auditory cognitive neuroscience.

In the future, it is likely that also in MMN studies the stimulus material will include elements of real music instead of only isolated sounds or repetitive computer-generated sound sequences. Even if this sounds implausible, there are possibilities already available that enable such studies. One means of meeting this challenge is offered by music information retrieval (MIR) technology. Using a MIR toolbox (Lartillot and Toiviainen, 2007) it is possible to identify acoustical and musical events (sounds or sound sequences) and code with trigger pulses any sound of interest, be it repetitive or surprising in its context. This can be done before or after an experiment to recorded music or after the experiment to a music recording based on live performance during a study. MIR-based ERP analyses were already conducted by Poikonen et al. (2016) for sounds that had the largest computational values related to timbre, harmony, and dynamics. After averaging the ERPs following each of these sound categories, N100 and P200 responses were computed and compared between three different compositions. More recently, Haumann et al. (2021) elaborated and further tested the feasibility of such analyses with different musical excerpts, again with focus on P1-N1-P2 responses.

Naturally, it should be considered that to utilize MIR-based analysis in MMN studies, it is necessary to include some repetitive sound features in the music excerpts. However, this repetitiveness can also be interpreted in abstract terms, such that sounds to be used as one category in the analyses differ from each other in their exact acoustical features but also simultaneously form a distinct category of the other sounds of a given musical excerpt up to a sufficient degree (e.g., instrumental sounds that form a category “novel instrument” even if they differ from each other acoustically). By careful behavioral screening of the participants’ cognitive, emotional, and aesthetic ratings of the sounds as by Sarasso et al. (2022), we can additionally categorize the sounds and subsequent ERP/MMN responses not only based on their acoustical or musical features but also by their perceptual loadings. By these procedures, we can continue developing the MMN study paradigms on sounds as part of music and not merely on sounds as such.

## Data availability statement

The original contributions presented in this study are included in the article/supplementary material, further inquiries can be directed to the corresponding author.

## Author contributions

The author confirms being the sole contributor of this work and has approved it for publication.
